# Room-temperature 2D semiconductor activated vertical-cavity surface-emitting lasers

**DOI:** 10.1038/s41467-017-00743-w

**Published:** 2017-09-14

**Authors:** Jingzhi Shang, Chunxiao Cong, Zilong Wang, Namphung Peimyoo, Lishu Wu, Chenji Zou, Yu Chen, Xin Yu Chin, Jianpu Wang, Cesare Soci, Wei Huang, Ting Yu

**Affiliations:** 1 NanjingTech-NTU Joint Center of Research and Development, Nanjing Tech University, Nanjing, 211816 China; 2 Division of Physics and Applied Physics, School of Physical and Mathematical Sciences, Nanyang Technological University, Singapore, 637371 Singapore; 3 State Key Laboratory of ASIC & System, School of Information Science and Technology, Fudan University, Shanghai, 200433 China; 4 Key Laboratory of Flexible Electronics (KLOFE) and Institute of Advanced Materials (IAM), Jiangsu National Synergetic Innovation Center for Advanced Materials (SICAM), Nanjing Tech University (NanjingTech), 30 South Puzhu Road, Nanjing, 211816 China; 5 Key Laboratory for Organic Electronics and Information Displays (KLOEID) and Institute of Advanced Materials (IAM), SICAM, Nanjing University of Posts and Telecommunications, 9 Wenyuan Road, Nanjing, 210023 China

## Abstract

Two-dimensional (2D) semiconductors are opening a new platform for revitalizing widely spread optoelectronic applications. The realisation of room-temperature vertical 2D lasing from monolayer semiconductors is fundamentally interesting and highly desired for appealing on-chip laser applications such as optical interconnects and supercomputing. Here, we present room-temperature low-threshold lasing from 2D semiconductor activated vertical-cavity surface-emitting lasers (VCSELs) under continuous-wave pumping. 2D lasing is achieved from a 2D semiconductor. Structurally, dielectric oxides were used to construct the half-wavelength-thick cavity and distributed Bragg reflectors, in favour of single-mode operation and ultralow optical loss; in the cavity centre, the direct-bandgap monolayer WS_2_ was embedded as the gain medium, compatible with the planar VCSEL configuration and the monolithic integration technology. This work demonstrates 2D semiconductor activated VCSELs with desirable emission characteristics, which represents a major step towards practical optoelectronic applications of 2D semiconductor lasers.

## Abstract (web summary)

Two-dimensional materials have recently emerged as interesting materials for optoelectronic applications. Here, Shang et al. demonstrate two-dimensional semiconductor activated vertical-cavity surface-emitting lasers where both the gain material and the lasing characteristics are two-dimensional.

## Introduction

Nowadays, semiconductor lasers have been extensively used in many practical applications such as fibre-optic communications, optical storage and laser spectroscopy. Developing the new semiconductor lasers via selecting gain media or cavity structures is attractive for both laser physics and potential applications. In particular, monolayer transition metal dichalcogenides (TMDs) of MoS_2_, WS_2_, MoSe_2_ and WSe_2_ with direct band gaps present much stronger excitonic emission than their thicker layers and bulks, which is triggering the investigation of monolayer lasing devices^1, 2^. Point- and edge-emitting lasers based on mono-^1, 2^ and few-layer^3^ TMDs have been successfully fabricated by use of a photonic crystal nanocavity^1^, a microdisk resonator^2^ and a coupled microdisk-microsphere cavity^3^. Strong light-matter coupling^4–6^ and photoluminescence (PL) enhancement^7^ have also been observed in two-dimensional (2D) semiconductor microcavities. These 2D semiconductors together with diverse photonic architectures are providing a new material playground for studying unique fundamental properties and developing cutting-edge optoelectronic applications^8, 9^. For example, the potential on-chip integration of monolayer lasing devices will open up many opportunities for the next-generation optical interconnects^10^ and supercomputing^11^.

In general, to turn the spontaneous emission (SE) from a 2D semiconductor into lasing, the proper resonant cavity and reflecting interfaces are required such as photonic crystal nanocavity^1^, microdisk cavity^2^ and cavity-air/vacuum interfaces. Previously, in view of intense and nonblinking PL of monolayer WS_2_ together with its planar nature, we proposed to employ such 2D semiconductors as the active media to realise vertical-cavity surface-emitting lasers (VCSELs)^12^. Meanwhile, in most practical laser applications, room-temperature continuous-wave operation is required, favourably with low-threshold and single-mode features. Developing 2D semiconductor activated VCSELs (2DVLs) with these attractive merits are challenging. One of the major obstacles is the low luminescence quantum yields of these as-exfoliated monolayer semiconductors, typically ranging from 0.01 to 20%^2, 12–15^, which are smaller than representative internal quantum efficiencies^16, 17^ ( > 70%) of classic III–V semiconductors. Nevertheless, one prevailing advantage of 2D semiconductors as gain media in VCSELs is the facile integration into the ultimate thin cavity. In contrast, the gain media in most semiconductor VCSELs are required to be epitaxially grown on the lattice-matching supporting layers and the typical cavity lengths are still from few to tens of half-wavelength thicknessses^18^. Moreover, introducing Purcell effect by precisely positioning a 2D semiconductor at the antinode of the cavity standing wave along with the proper in-plane confinement is one effective strategy to greatly enhance the SE rate^19^. In particular, these monolayer TMDs in 2DVLs own high refractive indices (e.g., 4–7) around the spectral regions of their exciton emission bands^20^, which is good for formation of efficient optical confinement after introducing the surrounding cavity materials with low-refractive indices.

In this work, we demonstrate room-temperature 2D lasing from monolayer WS_2_ embedded in a half-wavelength-thick cavity, where a dominant single-mode has been obtained under low-power continuous-wave photoexcitation. The whole piece of monolayer WS_2_ has been employed as the gain medium and inserted right at the only antinode of the longitudinal cavity mode for the 2D lasing. Altogether with the selected working wavelength and cavity materials, particularly in view of quantum yield, absorbance and refractive-index contrast, ultralow lasing threshold has been obtained.

## Results

### Design and formation of a single-mode 2DVL

**Fig. 1:**
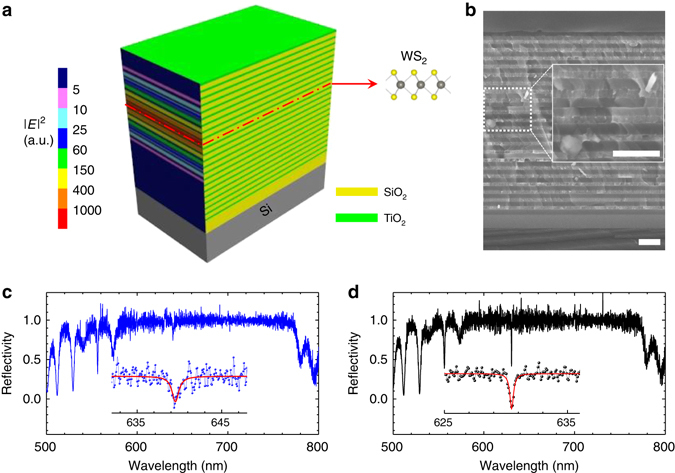
Structure of a 2D semiconductor embedded microcavity and its reflection. **a** Schematic of a 2D semiconductor embedded microcavity on the SiO_2_/Si substrate consisting of 12.5 pairs of bottom TiO_2_/SiO_2_ distributed Bragg reflectors (DBRs), half-wavelength-thick SiO_2_ cavity with embedded monolayer WS_2_ and 8.5 pairs of top DBRs. **b** Representative scanning electron microscope image of the cross-section. The *inset* shows the zoom-in image. *Scale bars* in **b** and its inset represent 500 nm. **c**, **d** Reflection spectra of monolayer WS_2_ embedded microcavity (in *blue*) and the blank microcavity region (in *black*) in the spectral range from 500 to 800 nm, respectively; The *insets* show the enlarged spectra together with the Lorentzian fits (in *red*) in the cavity-mode regions, respectively

The schematic structure of the 2DVL is shown in Fig. 1a, where the left side view presents the cross-sectional distribution of the electric field intensity of the cavity mode at 636.5 nm. The corresponding curve of electric field distribution is shown in Supplementary Fig. 1. Before the preparation of the bottom distributed Bragg reflectors (DBRs), one layer of SiO_2_ was pre-deposited to reduce the potential strain effect caused by the commercial 300 nm-SiO_2_/Si substrate. The bottom DBRs contains 12.5 pairs of quarter-wavelength-thick SiO_2_/TiO_2_ layers to ensure the reflectivity is above 99%. And then, the half-wavelength-thick cavity including two spacer layers of SiO_2_ and monolayer WS_2_ were fabricated, where the exfoliated monolayer WS_2_ was placed at the cavity centre via the dry transfer technique. The large difference of refractive indices between SiO_2_ and monolayer WS_2_ makes for strong optical confinement. After that, 8.5 pairs of top SiO_2_/TiO_2_ DBRs were deposited with a maximum reflectivity about 98%. Monolayer WS_2_ was selected here because of its relatively high quantum yield among the prevailing monolayer TMDs^2, 14^ and the exfoliated samples were used mainly due to its stable emission in small areas (e.g., typically several micrometres). Such a small area of gain medium is generally beneficial to active volume control and lateral confinement^19, 21^. Figure 1b shows a representative cross-sectional scanning electron microscope (SEM) image of a prepared sample and the inset is the enlarged image from the marked cavity region, where periodic structures with clear interfaces can be identified. The normal reflection spectrum has been measured for a monolayer WS_2_ embedded 2DVL, as shown in Fig. 1c. The high-reflectivity region of the stopband is broad, which covers the whole spectral range of the SE of monolayer WS_2_ and leads to the strong optical confinement. A dip appearing within the stopband, which corresponds to the cavity photon mode, can be clearly identified in the enlarged spectrum (the inset). It locates at 639.5 nm with a width of 1.0 ± 0.2 nm, indicating a typical quality factor *Q* of about 640. Moreover, the reflection spectrum in the blank cavity region (i.e., without monolayer WS_2_) is shown in Fig. 1d, where the overall profile is similar to that of the 2DVL but the dip narrows (i.e., 0.35 ± 0.05 nm) and shifts to the short wavelength by about 9 nm due to the lack of monolayer WS_2_. Accordingly, the *Q* for the blank region is about 1800 and the estimated refractive-index of monolayer WS_2_ is 5.6 in this spectral range, consistent with the previous report^20^. By contrast, the lower *Q* in the 2DVL is associated with the spectral broadening induced by monolayer WS_2_ insertion. Similarly, the reduction of *Q* factor from 8000 to 1300 after monolayer WSe_2_ transfer has also been observed in the monolayer WSe_2_ nanocavity lasers^1^. Hence, further investigation on the underlying broadening mechanisms and the better integration strategy of 2D active layers with photonic architectures would be very helpful to improve the performances of the rising 2D semiconductor lasers. Moreover, theoretical simulations of normal reflectivities of the blank microcavity and the designed 2DVL have been performed, which agree well with the observed reflection data (Supplementary Fig. 2 and Supplementary Note 1).

### Distinctive lasing features of a 2DVL

**Fig. 2:**
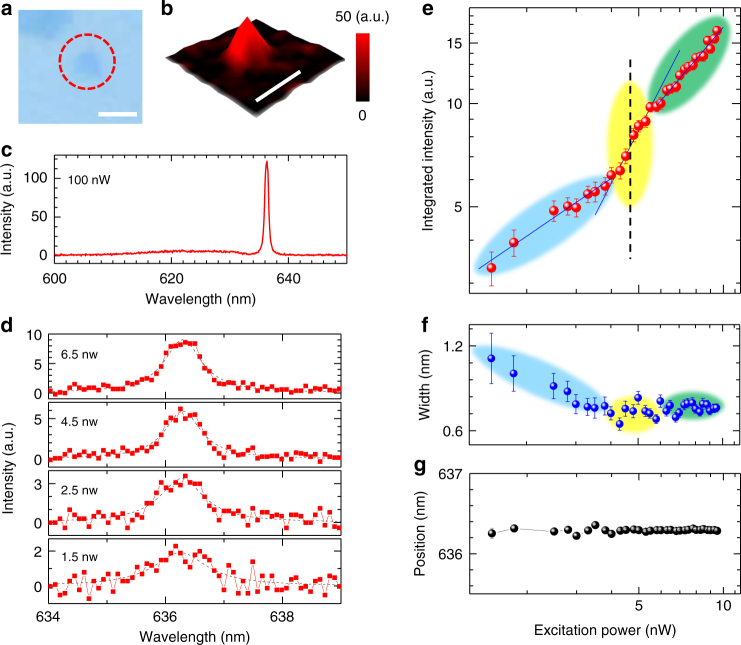
Room-temperature single-mode vertical lasing. **a** Optical image of an isolated monolayer WS_2_ on the half cavity marked by the *red dashed circle* and **b** spatially resolved photoluminescence (PL) intensity mapping of the complete microcavity sample (integrated in the spectral range from 635.5 to 637.5 nm). The *scale bars* represent 2 µm. **c** The PL spectrum collected at the sample centre and at the excitation power of 100 nW. **d** PL spectra collected at the sample centre under varied excitation powers  < 10 nW. The *grey dashed lines* are Lorentzian fits to the data. **e**–**g** PL output intensity, width and position as functions of excitation power, respectively. Error bars are extracted from Lorentzian fits. In **e**, three regions with different slopes are labelled as spontaneous emission (SE, *blue*), superlinear amplification (SLA, *yellow*) and lasing (*green*), respectively, where the *dashed black line* indicates the threshold region. The extracted slopes of SE, SLA and lasing are about 0.6, 1.6 and 1.0, respectively. With the increasing excitation power, the spectral width narrows **f**, while the position remains stable **g**

Figure 2a shows a monolayer WS_2_ sample on the half cavity, which owns a triangle-like shape and a lateral size of about 1.5 µm. After deposition of the other half cavity and top DBRs, the PL measurements were carried out at room-temperature, where a continuous-wave 532 nm laser was used for photoexcitation. The spatial imaging of PL intensity between 635.5 and 637.5 nm is shown in Fig. 2b. The peak intensity varies mainly due to the limitation of the laser spot size while the peak position and width are uniform across the sample (Supplementary Fig. 3). The representative PL spectrum (Fig. 2c) presents a sharp emission peak around 636.3 nm and a weak band due to leaked spontaneous emission (LSE). More specifically, Fig. 2d presents the steady-state PL spectra from this 2DVL sample centre at low-excitation powers and in the narrow spectral range from 634 to 639 nm, where the emission band grows and narrows with the increase in excitation power. The series of PL spectra at other excitation powers and/or in the broad spectral ranges are shown in Supplementary Figs. 4, 5. With the increasing excitation power, the evolution of the emission peak at 636.3 nm has been investigated. The log-scale plot of its integrated PL intensity as a function of excitation power is shown in Fig. 2e (i.e., light–light curve), where a kink appears together with two intersections around 4 and 6 nW, respectively. The rapid change of the intensity can also be identified in the linear plot (Supplementary Fig. 6). Three regions can be classified as SE, superlinear amplification (SLA) and lasing, respectively. The data in SE show a sublinear dependence, most probably due to the quenching of exciton emission by nonradiative decay channels such as exciton–exciton annihilation, consistent with its low quantum yield and previous reports^3, 14, 22, 23^. The superlinear behaviour presents in the SLA range and the nearly linear dependence fits well with the data in the lasing region. In a word, such a nonlinear response in the light-light curve is a strong signature of lasing behaviour. Note that, such sublinear to superlinear transition features have also been observed in microcavity lasers based on organic single crystals^24^, which are strongly correlated with the large excitonic effects of organic gain media being analogous to monolayer TMD cases.

Meanwhile, the corresponding peak widths at different excitation powers are shown in Fig. 2f, where the variation of peak width occurs. In details, the peak width drops during the transition from SE to SLA and the minimum width is found in the SLA region. After that, it remains stable at relatively high excitation powers. Similar trends of the peak narrowing have also been observed around threshold regions for other microcavity lasing systems^25, 26^ and recent 2D semiconductor lasers^1–3^, which is another fingerprint for the transition from SE to lasing. Besides, there is no obvious change in the peak position with the increasing excitation power (Fig. 2g), which is different from the typically observed blueshift for polariton lasing^27, 28^. Considering the transition region^29^ of SLA, the extracted lasing threshold is about 5 nW (i.e., corresponding to 0.44 W cm^−2^), comparable with the one of monolayer WSe_2_ lasing (1 W cm^−2^) obtained at low temperatures^1^ and much smaller than the reported thresholds of monolayer WS_2_ (i.e., 5–8 MW cm^−2^ at 10 K)^2^ and few-layer MoS_2_ (i.e., 5 µW at room-temperature)^3^. By analysis of the Purcell enhancement of our microcavity, the SE coupling factor is estimated to be 0.77 ± 0.1, which is in the same order of recently reported ones (i.e., 0.19–0.68)^1–3^. (Supplementary Note 2 and Supplementary Fig. 7) At higher excitation powers of 10–100 nW, the lasing emission shows the linearly increased intensity, stable width and position (Supplementary Fig. 8).

**Fig. 3:**
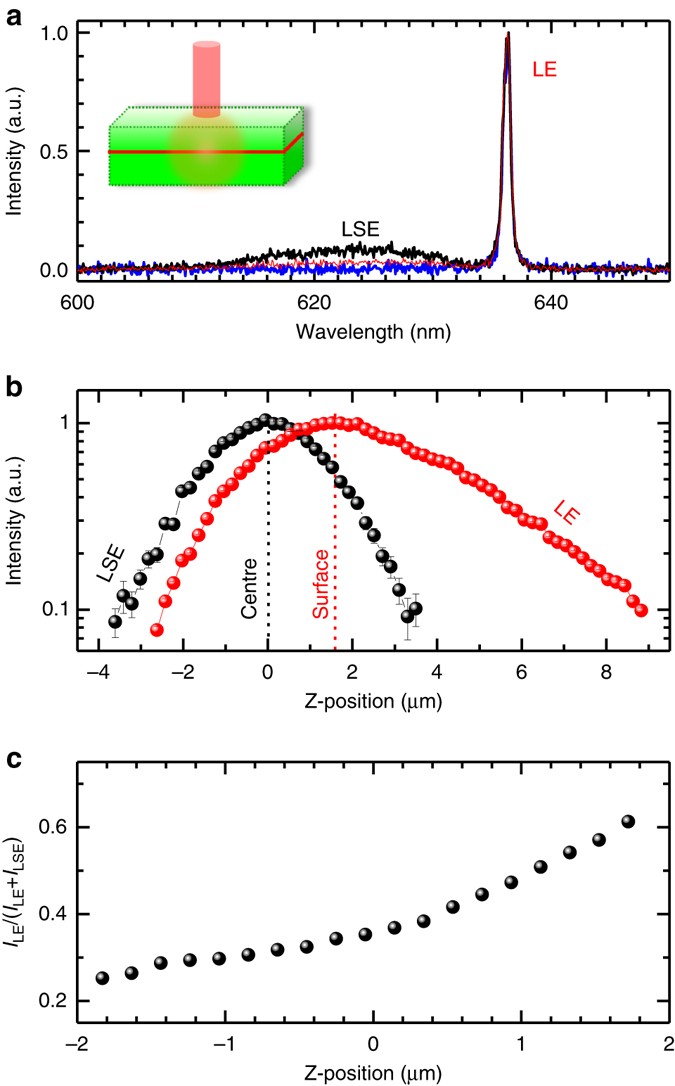
Confocal photoluminescence scanning along *Z*-direction. **a** Normalised photoluminescence spectra collected at the cavity centre (*black*), surface (*red*) and 5.1 µm (i.e., the distance above the cavity centre, *blue*), respectively. The used excitation power is 100 nW. The inset shows the emission schematic. **b** Comparison of normalised integrated intensities of the lasing emission (LE) and the leaked spontaneous emission band (LSE) at various *Z*-positions. *Error bars* are extracted from single and double Lorentzian fits to the confocal spectra. **c** The lasing intensity ratio to the detected total light output as a function of Z-position. The defined *Z*-direction is perpendicular to the sample surface. *I*_LE_ and *I*_LSE_ represent the integrated intensities of LE and LSE, respectively

Furthermore, the peculiar lasing features of the 2DVL have been recognised by confocal PL measurements along the Z-direction, i.e., perpendicular to the sample surface. With the confocal microscopic configuration, the signals near the focal plane are collected while the out-of-focus light is effectively excluded, which provides much cleaner signals at a sampling depth than the ones from the conventional wide-field microscope. The reference position is set to the location of the active medium of monolayer WS_2_, which is right at the cavity centre. With the lift of the focal plane from the centre, the remarkable change of the spectral profile appears as shown in Fig. 3a, where the lasing emission (LE) becomes predominant together with the significant reduction of the LSE proportion. The emission profiles can be reproduced by single or double Lorentian functions, dependent on the Z-position (Supplementary Fig. 9). The normalised integrated intensities of LE and LSE are shown in Fig. 3b, where the spatial intensity of LE presents three distinctive characters from those of LSE. First, the maximum intensity of LE appears at the 2DVL surface while the one of LSE presents at the cavity centre as expected. The distance between two maxima agrees well with the measured thickness of 1.7 ± 0.1 µm according to the cross-sectional SEM image (Fig. 1b). This feature is a clear fingerprint for the LE from the surface while most cavity photons (i.e., photons at the lasing frequency) inside the 2DVL are not detectable by the objective lens due to strong optical confinement or formation of standing waves. The inset in Fig. 3a illustrates the emission schematic, which is supported by our simulation results (Supplementary Fig. 10). Note that, the lasing light emitting from the VCSEL surface is well accepted^30–32^. Second, the asymmetric intensity profile has been observed for LE. It indicates that the LE is directional and preferred in the designed low-reflective direction (i.e., out of the top DBRs). If the emitted light randomly propagated in all directions, such an asymmetric profile would not be observed. For instance, the SE of a monolayer WS_2_ flake shows the symmetric profile (Supplementary Fig. 11). Meanwhile, the emission profile of LSE from the 2DVL is also symmetric and identical to that of a monolayer WS_2_ flake (Supplementary Fig. 11b), indicating the similar emission nature. Third, the intensity of LE along the vertical direction shows the significantly slower decay than those of LSE from the 2DVL and SE from the monolayer WS_2_, which indicates the less divergence and the better spatial stability, i.e., a hint of the presence of certain spatial coherence. In other words, the observed slower decay of intensity reflects the lasing light is spatially more stable and less diffuse than SE. In the effective focus range around the cavity centre, the lasing intensity ratio to the observed total intensity rises with the lifting Z-position as shown in Fig. 3c, which is between 0.25 and 0.61. The comparable lasing fraction has also been observed in the monolayer WSe_2_ nanocavity lasers^1^. In order to characterize this type of 2DVL more quantitatively, we introduce a relative external efficiency *η*_re_ = *I*_LM_/(*I*_LM_ + *I*_SM_), where *I*_LM_ and *I*_SM_ represent the measured maximum integrated intensities of LE and LSE, respectively. In our case, taking *I*_LM_ and *I*_SM_ at the surface and the cavity centre, respectively, the extracted *η*_re_ is 42.5%.

### Lasing in two dimensions

**Fig. 4:**
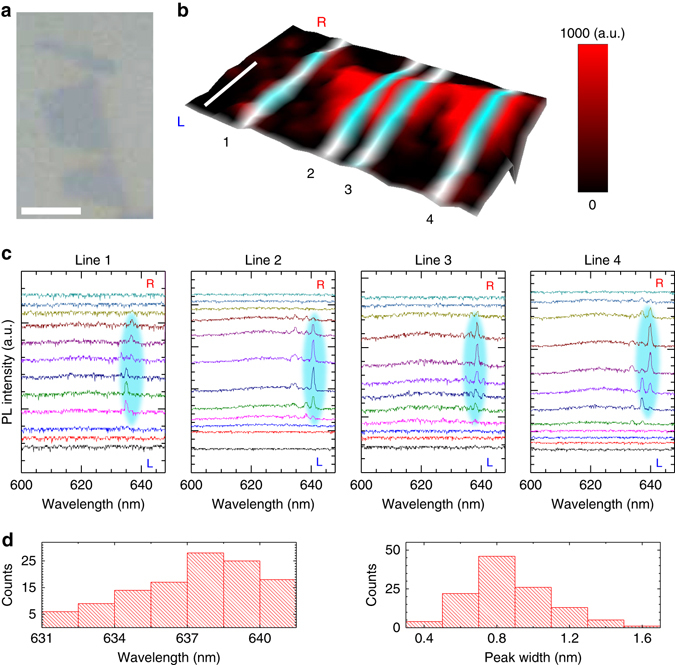
2D lasing from monolayer WS_2_ flakes. **a** Optical image of three 1L-WS_2_ flakes on the half cavity and **b** spatially resolved PL intensity image of the complete microcavity samples. The PL intensity is integrated from 600 to 645 nm. Line profile analysis was carried out along the marked lines. L and R indicate *left* and *right* sides, respectively. The *scale bars* represent 3 µm. **c** The series of PL spectra taken along four marked lines with a step size of 500 nm. The colour traces from *bottom* to *top* correspond to the spectra taken from *left* to *right* of each scanning line in **b**. The dominant lasing modes are highlighted in *cyan*. **d** Statistics of positions and widths of narrow emission bands in 67 spectra sampling from three flakes

To verify the spatially 2D lasing from the entire monolayer WS_2_ samples, another three pieces of nearly isolated monolayer WS_2_ flakes embedded in the same batch of microcavity have been examined. Figure 4a shows the optical image of three samples on the half cavity, which are few times larger than the laser spot size and thus can be well resolved. Figure 4b displays the three-dimensional plot of the spatially resolved PL intensity of the 2DVL samples taken at the excitation power of 100 nW, where the integration range is from 600 to 645 nm. The series of single spectra along four marked lines are presented in Fig. 4c. In Line 1, the emission spectra mainly show the single sharp peaks while more than one band appears in other lines, indicating the small active area is in favour of single-mode operation. The statistic results of 117 narrow emission bands in 67 spectra taken from three flakes are shown in Fig. 4d. Most peak positions of observed sharp bands are around 637 ± 4 nm and the peak widths are mainly around 0.8 ± 0.3 nm. The variations of peak position and width probably originate from the slight fluctuation of cavity thickness, for example, the thickness difference of 1 nm can result in the spectral shift of about 3 nm. Similar spatially non-uniform emission features have also been observed in conventional GaN-VCSELs^33^. Thus, further investigation can be improving the uniformity of cavity thickness in order to achieve the precise single-wavelength lasing across a large piece of sample.

## Discussion

The typical PL and absorption spectra of an exfoliated monolayer WS_2_ on quartz are dominated by A-exciton emission and absorption, respectively (Supplementary Fig. 12). Owing to the relatively low-quantum yield of monolayer WS_2_ at room temperature^2, 12, 14^, the wavelength selection of the designed cavity photon becomes critical to achieve the lasing here. In general, strong emission and low absorption at the designed wavelength are preferred, where the careful balancing is needed due to the spectral overlapping, especially in the case that the absorbance is comparable with the quantum yield. By comparing positions and profiles of absorption and emission bands, we notice that there is a slight Stokes shift between two spectra, which leads us to select the long-wavelength side of the emission peak. Technically, the emission wavelength of the cavity photon mode was tuned by adjusting the cavity thickness. In the case of nearby 635 nm, the lasing behaviours have been observed as discussed above. However, there is no clear lasing feature found for the sample with the designed cavity photon mode at 625 nm under the similar excitation conditions, which is mainly attributed to the relatively large absorption and the possible broadening of the cavity photon peak due to strong light-matter coupling and formation of microcavity polaritons^4–6^(Supplementary Fig. 13 and Supplementary Note 3). Besides, at the even longer wavelength (e.g., 650 nm), the bound exciton or defect emission may be involved, which requires further studies. Thus, the designed cavity photon between strong coupling and defect emission regions is an important factor to obtain the room-temperature lasing here. Further investigation will be the scalability and the electrical pumping of 2DVLs. On the one hand, the standard planar techniques of e-beam lithography and plasma etching can be used to realise the scalability in the size of the lasing area and the amount of 2DVL arrays on conventional silicon substrates. Meanwhile, the scaling of output power can also be actualised by improving the gain medium in the vertical direction such as multiple insertions of 2D semiconductors around the antinode regions and employment of multiple quantum wells containing 2D semiconductors and boron–nitride barriers^34^. During the scaling, additional attention needs to be paid to possible non-uniform effects of quantum yield and cavity modes. For industrial manufacturing purposes, the direct wafer-scale growth of 2D semiconductors on the half cavity substrates may be a better choice than the wet/dry transfer strategy of individual flakes in view that additional issues of contaminations, deformation, contact, scalability and compatibility with the integrated circuit technology are difficult to control in the latter case. On the other hand, electrically pumped 2DVL arrays could bring new opportunities for on-chip laser applications, where atomically thin emitting structures and improved quantum efficiency of electroluminescence will be critical to low-threshold and single-mode operation. To maintain the short cavity and reduce potential absorption losses, atomically thin layers^35^ rather than 2D semiconductor/bulk heterostructures^36^ are suggested to be used as the gain medium, which can be lateral or vertical *p*-*n* junctions prepared by in-situ growth, transfer or dual electrostatic gating^37^. The typical quantum yields of as-prepared monolayer semiconductors are low^2, 12–15^, which may hamper further realisation of electrically pumped 2DVLs. Nevertheless, such issues can be overcome with improved structural designs^34^ and material modifications^14^. For example, near-unity quantum yields of monolayer TMDs have been demonstrated by organic superacid treatments^14^. In practice, the electrically driven 2DVLs can be expected after further optimisation of resonant cavity structures and realisation of efficient electroluminescence from 2D semiconductor light-emitting diodes.

In summary, we have demonstrated room-temperature 2D single-mode dominant lasing from a monolayer semiconductor in an ultimately thin vertical cavity with continuous-wave ultralow-power photoexcitation. Our strategy to design 2DVLs for room-temperature lasing can be extended to other emerging 2D semiconductors and further investigation can shift to practical optoelectronic applications, for instance, fabrication of 2DVL arrays as low-power or large-area laser sources for optical interconnects, supercomputing, lighting panels and displays.

## Methods

### Fabrication of 2DVLs

The commercial SiO_2_ (300 nm)/Si (0.5 mm) wafer was used as the supporting substrate. High-impurity (99.9%) source materials of SiO_2_ and TiO_2_ powders were used to deposit the DBRs and cavities in a high vacuum chamber ( ~ 10^−6^ torr) by an e-beam evaporator (Cello, Ohmiker-50B).

### Optical characterisation

The PL spectra were measured using a confocal microscope system (WITec, Alpha 300) with a frequency doubled YAG 532 nm laser. The PL spectra in Fig. 2c, d were obtained by averaging ten acquisitions, where an integration time of 5 s was used for each. Except for the PL data in Fig. 2b and Supplementary Fig. 3, the excitation laser beam was focused onto the sample with a 50× objective (Nikon, LU Plan, NA = 0.55, WD = 10.1 mm) for all PL measurements, where the estimated spot size is 1.2 µm. Another 100× objective (Zeiss, NA = 0.9, WD = 0.31 mm) was used to perform the spatial PL mapping as shown in Fig. 2b for a better resolution. The normal reflection measurements^38^ were performed in the backscattering configuration of the same microscope system, where an integrated LED white-light source was vertically incident on the sample with a 5× objective lens (Zeiss, NA = 0.13, WD = 16.1 mm).

### Data availability

The data supporting the findings of this work are available from the corresponding authors.

## Electronic supplementary material

Supplementary Information
