# Supplementary material for: Room-temperature 2D semiconductor activated vertical-cavity surface-emitting lasers
Source: Nat Commun. 2017 Sep 14;8:543. doi: 10.1038/s41467-017-00743-w (PMC5599555; doi:10.1038/s41467-017-00743-w)
Supplement: Supplementary file 1 — Supplementary Information [file 41467_2017_743_MOESM1_ESM.pdf]

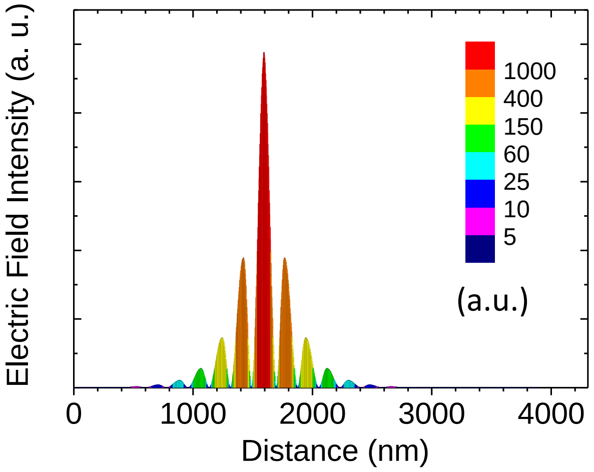

Supplementary Figure 1 | Electric-field distribution inside the microcavity. It shows the calculated electric-field distribution of the fundamental cavity mode inside the monolayer WS_2 activated vertical-cavity surface-emitting laser. The top surface of the sample is set as the reference position.

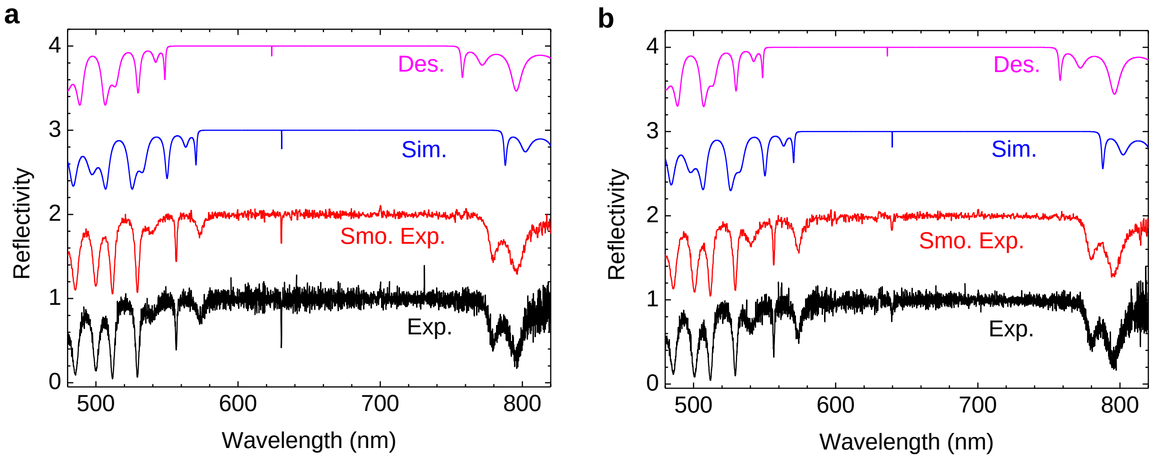

Supplementary Figure 2 | Characterization of cavity modes. (a) Experimentally measured and theoretically calculated reflection spectra of the blank microcavity region at normal incidence including experimental (Exp.), smoothed experimental (Smo. Exp.), simulated (Sim.) and as-designed (Des.) reflectivities. **(b)** Experimentally measured and theoretically calculated reflection spectra of the monolayer WS_2 embedded microcavity at normal incidence. The curves of Smo. Exp., Sim., and Des. are offset for the better illustration.

Supplementary Note 1. Technically, the signal/noise ratio of original experimental (Exp.) data can be improved through a standard signal smoothing process of the 50% percentile filtering (i.e. Smo. Exp.). The top curve shows the reflectivity of the as-designed (Des.) microcavity without considering practical imperfections induced during fabrication processes. The simulated (Sim.) reflectivity spectrum is obtained by taking the thickness deviations (< 6%) of deposition layers into account, in which the main features of the experimental reflectivity spectrum have been reproduced. Note that, the reflectivity of a practical cavity generally degrades referring to the ideal designed case due to the inevitable limitation of fabrication technologies such as the thickness fluctuation of each layer, material composition variations and unintentional defects. For example, in practice, a few more pairs of distributed Bragg reflectors (DBRs) are often required to be deposited to achieve the designed high reflectivity.

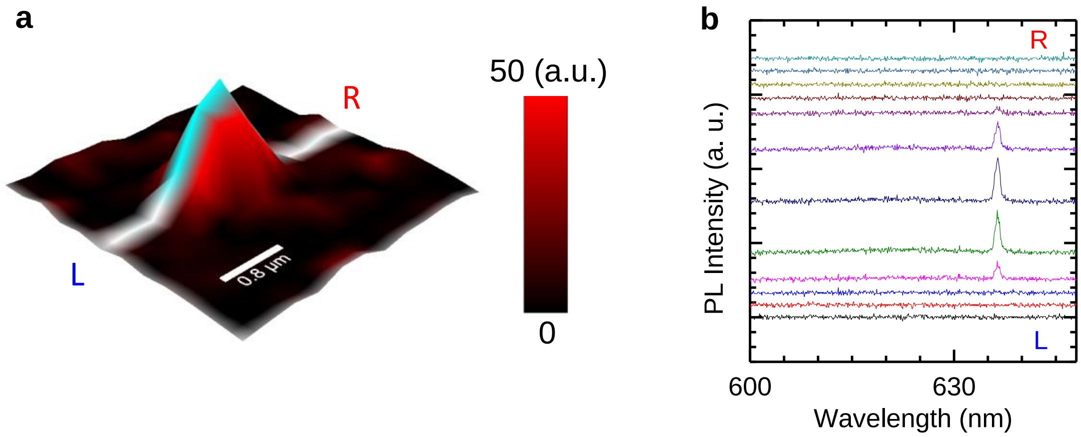

Supplementary Figure 3 | Spatial photoluminescence characterization of the microcavity. Zoom-in photoluminescence image **(a)** and the line profile analysis **(b)** across the sample. The curves from bottom to top were collected at sampling positions from left (L) to right (R) as indicated in **(a)** with a step size of 333 nm. The signal was collected with a 100× objective lens (NA = 0.9) at the excitation power of 78 nW.

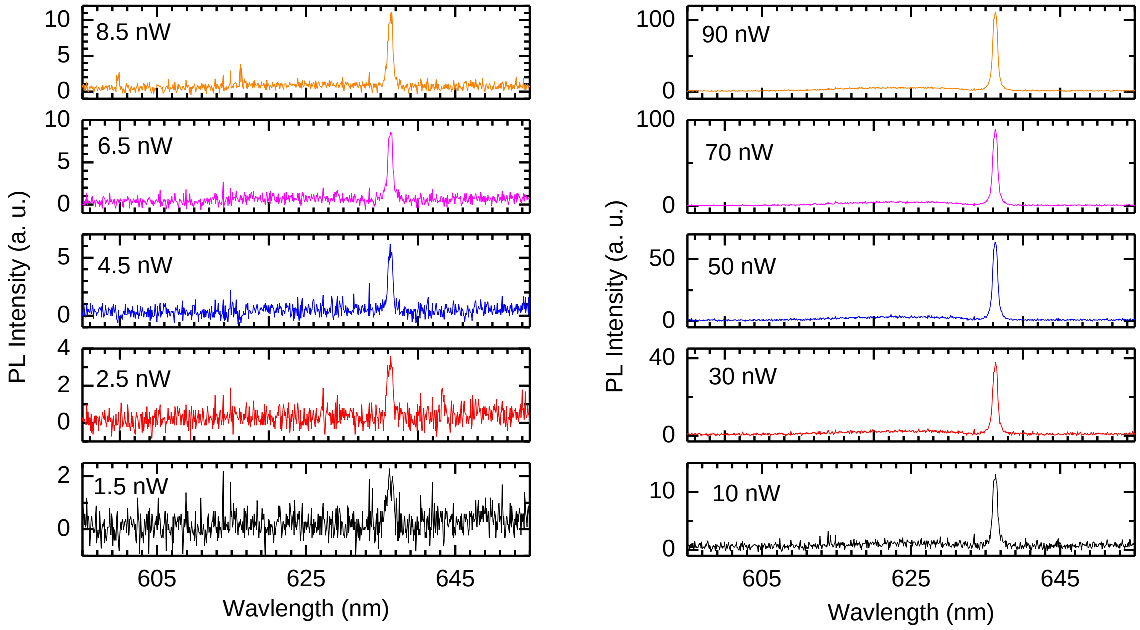

Supplementary Figure 4 | Wide-range photoluminescence spectra. Photoluminescence spectra collected at the sample centre under different excitation powers (i.e. 1.5-90 nW) in the spectral range from 595 to 655 nm.

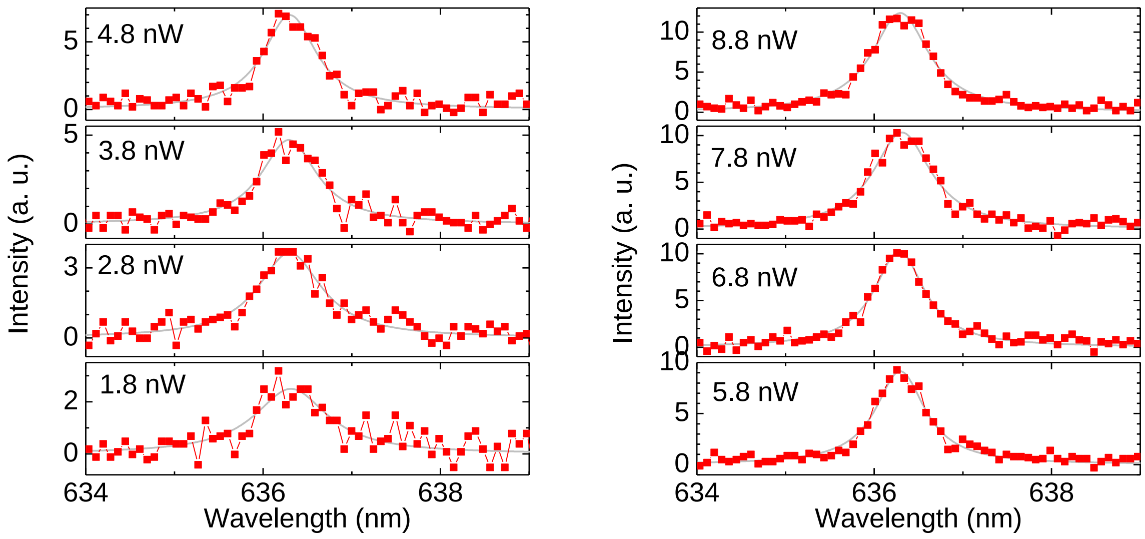

Supplementary Figure 5 | Zoom-in photoluminescence spectra. Photoluminescence spectra taken at other low excitation powers (< 10 nW) and their fit curves. Single Lorentzian function is used to fit the data and the fit curve is shown in grey.

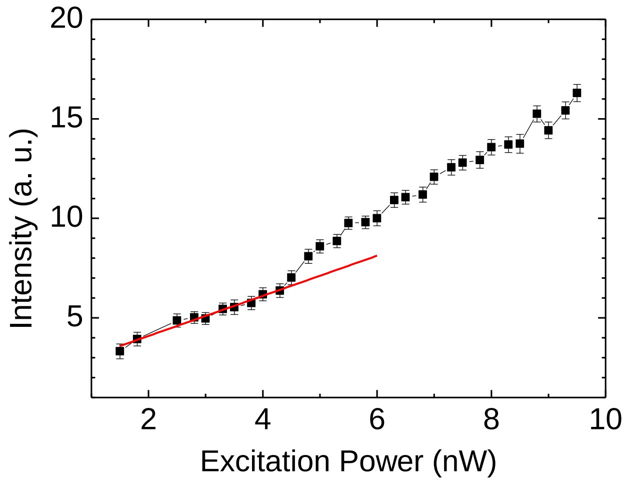

Supplementary Figure 6 | Photoluminescence intensities at ultralow excitation powers. Linear plot of the integrated intensity of the emission band at 636.3 nm as a function of excitation power. The red line is a guide to the eye.

.

Supplementary Note 2.

The spontaneous emission factor can also be estimated via analysis of the Purcell factor of spontaneous emission enhancement[1,2]. The following equation is used to estimate the Purcell factor,

$$F_{\mathrm{p}} = \frac{3}{4\pi^2}\frac{Q}{V_{\mathrm{c}}}\left(\frac{\lambda}{n}\right)^3 \quad (1)$$

In details, Q is the quality factor, which can be extracted by $\lambda/\Delta\lambda$. V_c is the active volume of cavity mode[3], which can be estimated based on the effective cavity length L_c, the active monolayer WS_2 area and the laser spot size (about 1.2 μm). In our case, the estimated L_c is 0.6 μm for the ideal sample, which will broaden in our practical devices due to structural imperfections such as growth thickness fluctuation and refractive index deviations. For the sampling location shown in Fig. 2, the estimated Q is 570 ±100 and the estimated V_c is 1.1 ± 0.4 μm^3 after considering the possible structural imperfections. When Q = 570 and V_c = 1.1 μm^3, the calculated F_p and $\beta = F_p/(1+F_p)$ are 3.4 and 0.77, respectively. If considering the variations of V_c and Q, the estimated β is 0.77±0.1 in our case.

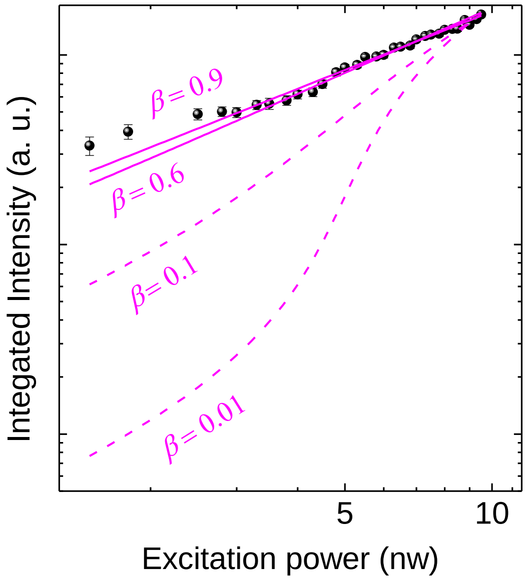

Supplementary Figure 7 | Data analysis of the light-light curve. According to microcavity rate equations[2], four simulation curves with different β values to compare with the experimental data are presented. Obviously, two curves with β = 0.1 and 0.01 are not suitable here. In contrast, above the initial sublinear region, our data can be nearly reproduced with larger spontaneous coupling factors β between 0.6 and 0.9 although the experimental errors hamper further precise fitting. Overall, this is consistent with the analysis based on Purcell enhancement above. Besides, the deviation of the curve fitting at low excitation powers is possibly because both large excitonic effects and optical losses by non-radiative decay processes were not considered in the simulation. The precise rate of non-radiative decay in monolayer WS_2 remains unclear at present but typically induces a larger β (refs.4 and 5). Further ultrafast spectroscopic studies on non-radiative carrier dynamics of monolayer WS_2 and theoretical reconsideration of large excitonic effects of 2D semiconductors on rate equations may help to fully address the experimental observations.

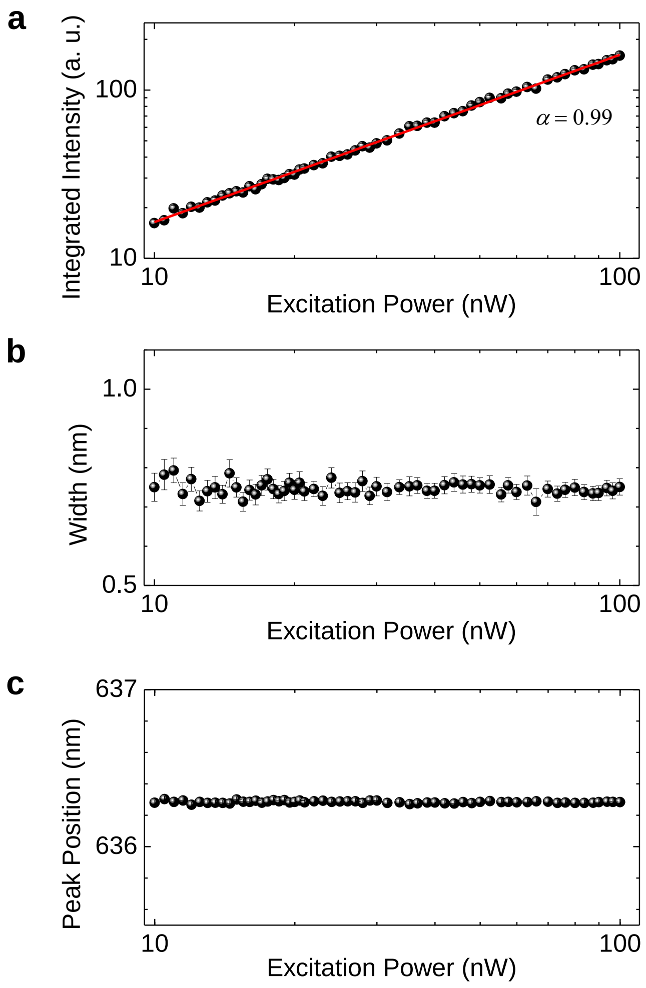

Supplementary Figure 8 | Photoluminescence characteristics at excitation powers above 10 nW. (a-c) Integrated intensity, width and peak position as functions of the excitation power between 10 and 100 nW, repectively. The red line in **(a)** shows the linear fit curve with a slope of $\alpha = 0.99 \pm 0.01$.

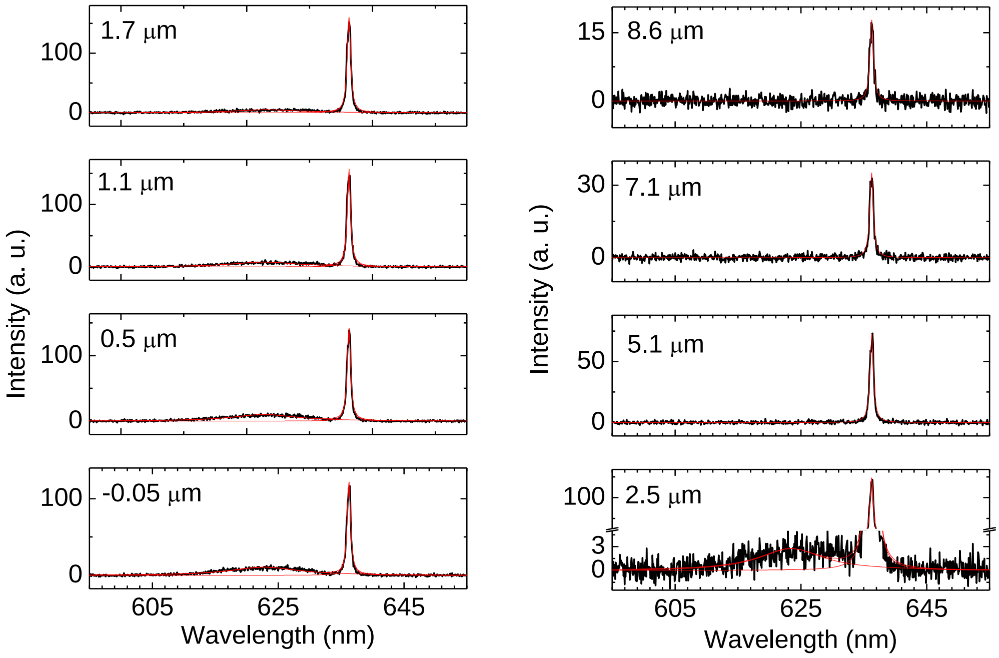

Supplementary Figure 9 | Photoluminescence evolution along the vertical direction. Photoluminescence spectra (in black) and their fit curves (in red) at different Z positions, where double and single Lorentzian functions (in red) are used to fit the data in the ranges of -0.05-2.5μm and 5.1-8.6μm, respectively. For the curve collected at 2.5μm, the Y scale is broken into two regions in order to show the fit details.

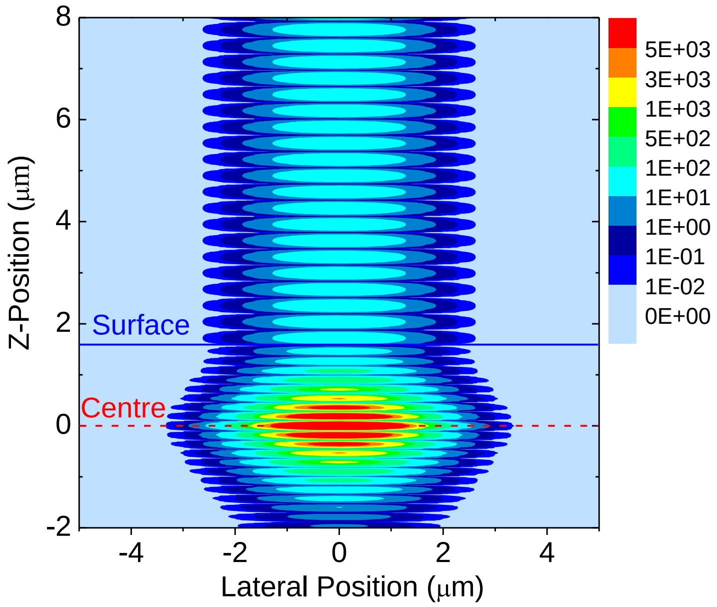

Supplementary Figure 10 | Simulated electric-field distribution under photoexcitation. The simulated output of the lasing component of the used 2D semiconductor activated vertical-surface emitting-lasers (2DVL) under a laser beam excitation with a Gaussian width of 1.2 μm. The colour bar represents the electric field intensity. Note that, most cavity photons under the 2DVL surface form the standing wave and almost remain inside the cavity. Hence, most internal cavity photons cannot be detected by the objective lens. In contrast, the lasing component around the surface can be most effectively collected during the confocal micro-photoluminescence measurements along the Z-direction. With raising or lowering the objective lens away from the surface, the defocus on the surface will lead to the decrease of the detected lasing signals. As a result, the maximum lasing intensity was observed close to the surface.

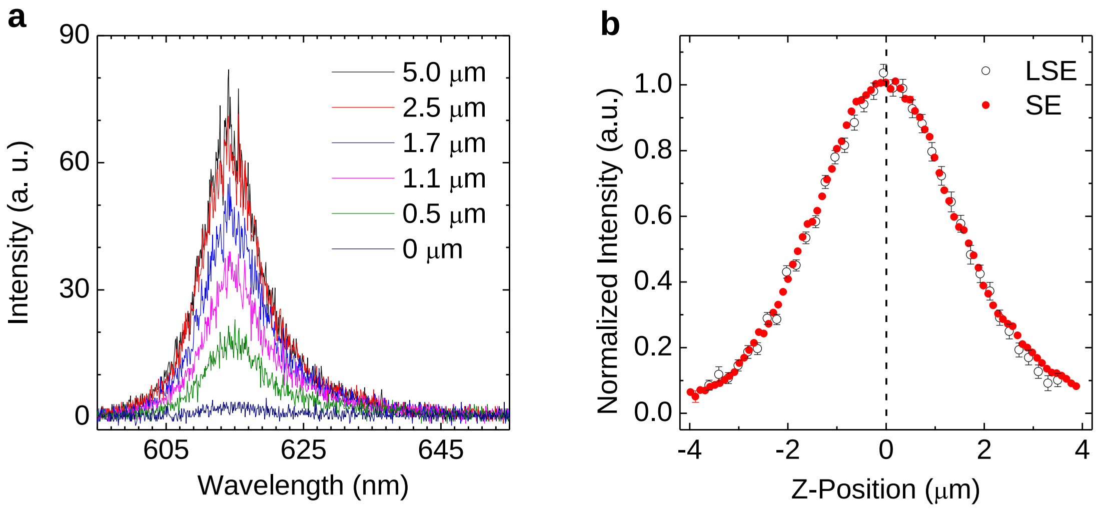

Supplementary Figure 11 | Confocal photoluminescence of monolayer WS_2 and its comparison with the lasing band. **(a)** Spontaneous emission spectra of a monolayer WS_2 flake taken along the Z direction (i.e. vertical to the sample surface). **(b)** Normalized integrated intensities of spontaneous emission (SE) from monolayer WS_2 and the leaked spontaneous emission (LSE) from monolayer WS_2 embedded microcavity (i.e. the same data shown in Fig. 3b) versus Z position. The SE and LSE show the similar intensity profiles, consistent with the same spontaneous emission nature in both bases. The extracted full width at half maximum is about 3.5 μm, which also reflects the depth of field of the used objective lens.

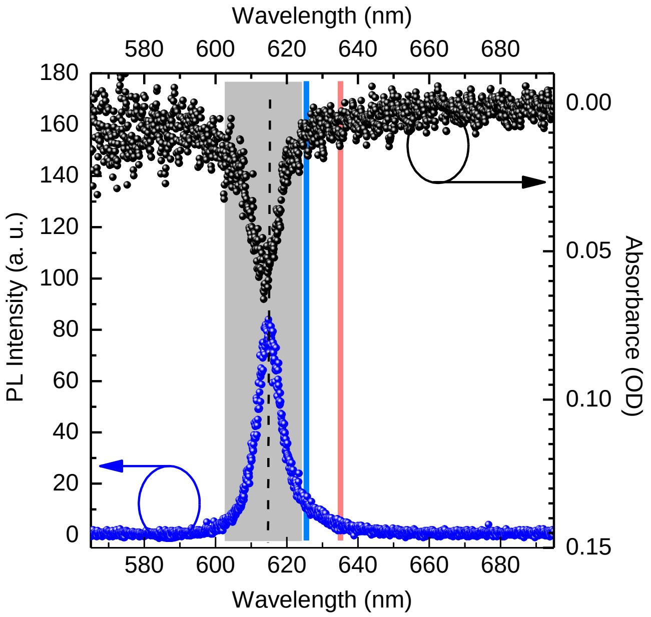

Supplementary Figure 12 | Selection of the wavelength of cavity mode. Photoluminescence emission and absorption spectra of monolayer WS_2 on quartz are shown in the lower and upper panels, respectively. Two marked wavelengths as indicated by red and blue lines are selected as the designed cavity photon modes for two sets of microcavity devices as shown in Figs. 2-4 and Supplementary Fig. 13, respectively. The dominant exciton emission and absorption bands present in the grey region, where the dashed black line indicates the emission peak position.

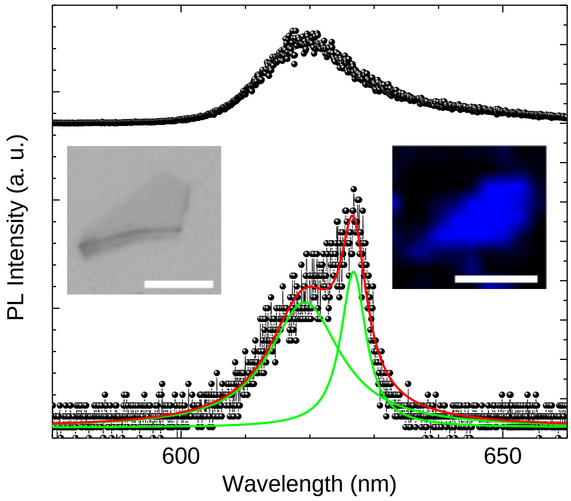

Supplementary Figure 13 | Characterization of a monolayer WS_2 embedded microcavity with a cavity mode of about 625 nm. Photoluminescence spectra from the sample on the half cavity (top) and within the full designed cavity (bottom). The insets are the optical image (left) of the sample on the half cavity and the photoluminescence mapping (right) of the complete sample. The designed cavity photon mode is at 625 nm and the excitation power is 100 nw. The scale bars in both images represent 5 µm.

Supplementary Note 3. This sample has the high reflectivity DBRs to confine the cavity photon, being similar to the 2DVL sample operating around 636.3 nm. The photoluminescence spectra of monolayer WS_2 inside the complete microcavity can be fit by two Lorentzian functions, which are assigned to the upper and lower polariton bands, being similar to several recent studies[6-8]. The overall fit curve and two fitting components are shown in red and green, respectively. The energy difference between two bands is 24.3 meV. Under the similar excitation conditions, there is no clear lasing behaviour observed. A recent study[7] has shown that the linewidth of LPB significantly broadens in the strong coupling regime, which will hinder to obtain the narrow lasing mode. Here, the leakage upper polariton band tends to merge with the LPB when the designed cavity photon energy is close to the dominant exciton emission (the grey region in Supplementary Fig. 12), i.e. in the strong light-matter coupling regime. In the case of 625 nm, the Rabi splitting energy ($\hbar\Omega = 2g_0$, g_0 is the coupling rate) should be smaller than the mentioned energy difference above and thus g_0 is less than 12.2 meV. In the 2DVL samples with the cavity photon wavelength of 636.3 nm, the typical separation between cavity photon and exciton band is about 45 meV, which is much larger than g_0. Thus, the device at 636.3 nm is working in the region where two emission bands behave like bare excitons and cavity photons[9].

Supplementary References

1 Purcell, E. M. Spontaneous emission probabilities at radio frequencies. *Phys. Rev.* **69**, 681-681 (1946).

2 Yamamoto, Y., Machida, S. & Bjork, G. Microcavity Semiconductor-Laser with Enhanced Spontaneous Emission. *Phys. Rev. A* **44**, 657-668 (1991).

3 Coldren, L. A., Temkin, H., Wilmsen, C. W. Vertical-Cavity Surface-Emitting Lasers: Design, Fabrication, Characterization, and Applications, Cambridge University Press, 68-74, 2001.

4 Bjork, G., Karlsson, A. & Yamamoto, Y. Definition of a laser threshold. *Phys. Rev. A* **50**, 1675-1680 (1994).

5. Wu, S. F. *et al.* Monolayer semiconductor nanocavity lasers with ultralow thresholds. *Nature* **520**, 69-72 (2015).

6 Liu, X. Z. *et al.* Strong light-matter coupling in two-dimensional atomic crystals. *Nat. Photonics* **9**, 30-34 (2015).

7 Dufferwiel, S. *et al.* Exciton-polaritons in van der Waals heterostructures embedded in tunable microcavities. *Nat. Commun.* **6**, 8579 (2015).
8 Flatten, L. C. *et al.* Room-temperature exciton-polaritons with two-dimensional WS_2. *Sci. Rep.* **6,** 33134 (2016).
9 Deng, H., Haug, H. & Yamamoto, Y. Exciton-polariton Bose-Einstein condensation. *Rev. Mod. Phys.* **82**, 1489-1537 (2010).
